# Comparison of the Y-pouch orthotopic neobladder and the Studer technique after radical cystectomy: surgical and functional outcomes from a single-center series

**DOI:** 10.1186/s12957-023-03112-8

**Published:** 2023-07-22

**Authors:** Sarayuth Boonchai, Monthira Tanthanuch, Tanan Bejrananda

**Affiliations:** Division of Urology, Department of Surgery, Faculty of Medicine, Prince of Songkla University, Hatyai, Songkhla, Thailand

**Keywords:** Radical cystectomy, Studer neobladder, Y-pouch neobladder, Bladder cancer, Orthotopic neobladder

## Abstract

**Background:**

To explore a method of constructing an orthotopic ileal neobladder (ONB) in the Y-pouch configuration. We describe the steps followed to create the Y-pouch ileal orthotopic neobladder (ONB) and compared the perioperative, functional, and urodynamics outcomes with the Studer neobladder technique.

**Methods:**

A retrospective cohort study of 90 bladder cancer patients, who received open radical cystectomy with the ONB performed at a hospital from June 2009 to May 2020. These patients were divided into two groups—the Y-pouch and the Studer neobladder groups. Perioperative, functional outcome, complication, renal function data outcomes, and pressure–volume study were used to evaluate the treatment outcomes after a radical cystectomy.

**Results:**

Ninety patients (54 Studer and 36 Y-pouch neobladder) were enrolled. The median patient age was 62.6 (± 11) years. The mean operative time for the Studer technique was 290 (242.5–350) min, and the Y-pouch technique was 300 (271.2–335) min) (*p* = 0.826). At 30 days postoperatively, the Clavien-Dindo classification of surgical complications revealed grade-2 urinary infections in two patients (5.6%) and six patients (11.1%) for the Y-pouch and Studer techniques, respectively. Intermediate complications (30–90 days) were reported in 4 (11.1%) and 18 patients (44.4%) in the Y-pouch and the Studer techniques, respectively (*p* = 0.062). In the urodynamics study (UDS), the Y-pouch group had a mean postvoid residual volume of 20 mL and Studer of 40 ml (*p* = 0.06). A mean capacity of 462 (380–600) mL compares to the Studer neobladder group with 495 (400–628) mL. The average mean compliance of the Studer group was 35.5 (28–52) ml/cm H_2_O and 33 (30–43) ml/cm H_2_O for Y pouch, and most patients had > 30 ml/cm H_2_O compliance (80/90 patients).

**Conclusions:**

The Y-pouch neobladder technique in an RC with an orthotopic neobladder provides perioperative and functional outcomes compared to those of the Studer orthotopic neobladder resulting in similar intermediate-term. Therefore, the Y-pouch ileal neobladder is both feasible and safe to be used as a standard neobladder technique for urinary diversion in patients with bladder cancer undergoing radical cystectomy and needs confirmation with long-term results.

## Introduction

A radical cystectomy (RC) is an effective method for the treatment of invasive bladder cancer, with an ileal orthotopic neobladder (ONB) reconstruction being an ileal method of urinary diversion [1, 2]. An orthotopic neobladder should enable normal bladder function, keep adequate capacity at low pressure, create a continence mechanism, and prevent upper urinary tract dilatation via an antireflux mechanism [3].

Initially, as described by Camey, a small tubular bowel segment has been used as a bladder substitute for the creation of an ONB and a tubular bowel segment anastomose is performed to the urethra directly without detubularization [4]. Subsequently, many different types of orthotopic neobladders using various gastrointestinal segments (ileum, ileo-colon, colon, sigmoid colon) have been reported [5–7]. Nowadays, intestinal detubularization plays a major role in constructing an adequate capacity reservoir with low pressure. While the most popular technique has been the one reported by Studer et al., who created a bladder substitute using a detubularized ileal pouch after an RC [8], in the MIS era, simple novel intracorporeal techniques have been described of which the Y pouch may be the technique of interest [9–11].

The aim of the study was to explore a new method of constructing an ONB in the Y-pouch configuration. Focusing on patients treated with open RC, we describe the steps followed to create the Y-pouch IONB. We also compared the perioperative and functional outcomes of this approach with those of the Studer neobladder technique.

## Patients and methods

### Patient selection

This study was a retrospective analysis of the clinical data of 90 patients with bladder cancer, who underwent an RC with ONB at a university hospital, from June 2009 to May 2020. Among them, 36 were treated using the novel Y-pouch technique and 54 were treated using the neobladder technique. Both the cystectomy and reconstruction with a Y-pouch and Studer techniques were performed using open surgery. Their clinical preoperative, perioperative, and also postoperative data were reviewed. The decision-making process for selecting the method of establishing the neobladder involved a comprehensive evaluation based on various clinical indicators and principles of surgical protocol development. The selection bias method of establishing the orthotopic neobladder is determined according to time bias, with more including the Studer technique in the early period and the Y-pouch technique in the late period. All cases were performed under highly experienced urologists. Also, ERAS management and cystostomy were not applied routinely in all cases.

### Surgical technique: The Y-pouch neobladder

**Fig. 1:**
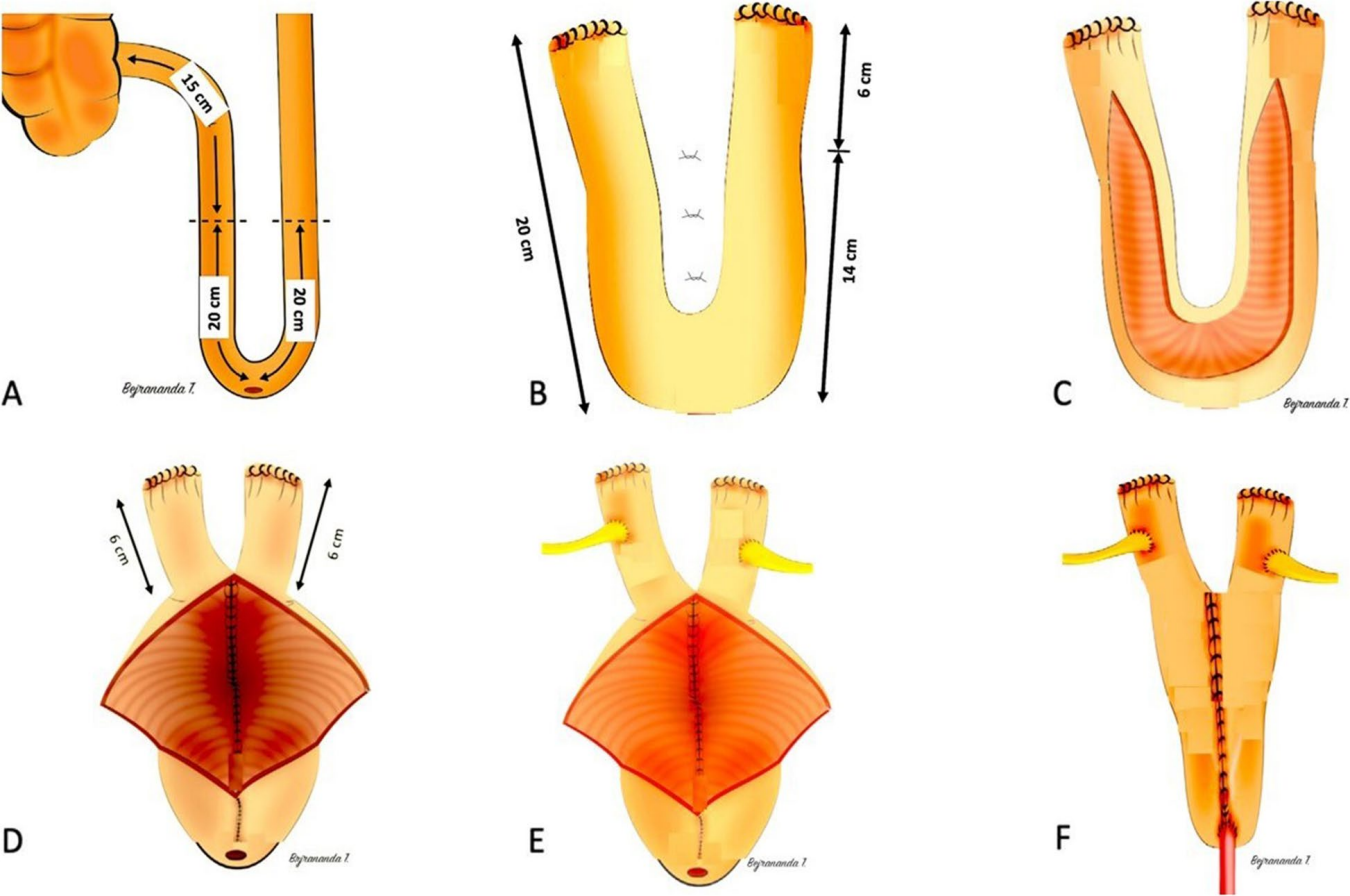
Surgical steps in the creation of the Y-pouch neobladder. **A** Harvest of 40 cm of ileal segment. **B** Creation of Y-pouch configuration. **C** Separation of the ileal segment at the antimesenteric border for detubularization. **D** Continuous suturing of medial edges of the ileal loop. **E** Anastomosis of uretero-neobladder. **F** Suturing of the anterior wall of the neobladder and performance of urethroileal anastomosis over a transurethral 20-French catheter

A 40-cm ileal segment was harvested, the starting point of which was selected at 15 cm from the ileocecal junction (Fig. 1A). The harvested bowel was cleaned and sutured at the proximal and distal ends with 3–0 polyglactin. The ileal intestinal continuity was maintained using a side-to-side anastomosis with an endoGIA stapler. A Y-configuration of the harvested bowel segment was created, with the set point for the bottom being at 20 cm. Next, the medial sides of the ileal loop were fixed using interrupted sutures of 3–0 polyglactin along a 14-cm segment from the bottom (Fig. 1B). The 28-cm ileal segment was cut at the antimesenteric border for detubularization in a Y configuration with 6 cm of both Y-limb ends being spared (Fig. 1C). The medial rims of the ileal loop were sutured with 3–0 polyglactin interlockings running through (Fig. 1D). Both the ureters were cut, spatulated medially, and then anastomosed end-to-side to both distal parts of the Y limbs of the neobladder with appropriate feeding tube stents or JJ stents using the Bricker technique without an antireflux mechanism (Fig. 1E). After this stage, a Y configuration was formed, the lateral rim of the ileal loop a running suture using 3–0 polyglactin was made, and stomatization performed in the lower part of the reservoir, in order to create a urethroileal anastomosis. The ureteral stents were fixed to the ileal mucosa and taken out of the pouch by stabbing the anterior wall of the pouch with or without a cystostomy. The urethroileal anastomosis was performed using a transurethral 20-French catheter with six 3–0 polyglactin sutures. Finally, a 10-Fr Jackson-Pratt drain was placed in the pelvic cavity (Fig. 1F).

### Surgical technique: The Studer neobladder

For the Studer neobladder technique, after performing radical cystectomy and pelvic lymphadenectomy, an orthotopic diversion is created by isolating a 50–60-cm long segment of the ileum. The segment is located 25 cm proximal to the ileocaecal valve to avoid the risk of vitamin B12 malabsorption or bile acid-induced diarrhea. The distal end of the ileal segment, measuring 40–44 cm, is opened along its antimesenteric border, while the proximal 15 cm remains tubularized (referred to as the “afferent limb”). The ureters are then individually connected to the afferent tubular ileal segment in an end-to-side manner. This part of the procedure is identical to the traditional Studer technique, until the posterior surface of the bladder substitute is formed, as shown in Fig. 1. Small bowel continuity is established using mechanical staplers and a running 3–0 Vycril seromuscular suture in a latero-lateral fashion. The reservoir reconstruction is performed manually using a series of continuous 3–0 Monocryl sutures on the luminal side and 3–0 Vycril on the serosal side [12].

### Follow-up

We removed the ureteric stent on the postoperative 5th day and administered a single-shot IV third-generation cephalosporin before stent removal. The patient was discharged after the Jackson-Pratt drain was removed or after the removal of the Foley catheter on postoperative days 28 with proof of no leakage from the cystogram. At 1 month, the first postoperative evaluation was performed and self-bladder irrigation was started. After the treatment, we carried out postoperative reviews of the patients at 1 month, and then at 3, 6, and 12 months, and at least yearly thereafter. The review included a clinical examination, an assessment of the renal function (i.e., serum creatinine levels, and estimated glomerular filtration rates), and routine laboratory values as well as computerized tomography scans of the chest and abdomino-pelvis scan or KUB ultrasound.

### Parameters and endpoint

The observation indicators of this study were as follows. First, the baseline parameters of the patients. Second, the perioperative and postoperative outcome observation indicators included early and late complications, daytime continence, nighttime continence, continence status, capacity, residual urine, and the Clavien-Dindo classification [13]. Continence was self-reported by the patient during the follow-up visits. If the patient used more than 1 pad per day, this was considered urinary incontinence. Nighttime continence is defined as using 0 or 1 safety pad per night. Sexual potency or the sexual bother domain is assessed with a single question. Potency was defined as the ability to achieve and maintain satisfactory erections or firm enough for sexual activity or sexual intercourse.

The capacity of the neobladder was measured by ultrasound or cystography under fluoroscopy. The outcomes were compared between the Y-pouch and the standard Studer neobladder groups [8].

### Urodynamics study (UDS)

All urodynamics study (UDS) measurements were analyzed at 12 months following the surgery. The patients are placed in a sitting position. Noninvasive uroflowmetry and postvoid residual volume (PVR) were assessed at the beginning of the procedure. The change in volume per increase in pressure during the filling phase was used to calculate compliance (ml/cm H_2_O). Provocative testing (Valsalva maneuver and coughing) was used to assess stress urinary incontinence and abdominal leak-point pressure (ALPP) at maximum cystometric capacity.

### Statistical analysis

The R program version 4.0.1 was used for the statistical analysis of our data. All the quantitative data were represented by a mean ± SD or an interquartile range (IQR), and comparisons between 2 different time points (change of eGFR and creatinine for each subject over a 12-month period) were performed using paired *t* tests or Wilcoxon tests. The count data were represented by examples (*n*) and frequencies (%), and Pearson’s chi-square test or Fisher’s exact test was used for intergroup comparisons. *P* < 0.05 was considered to indicate statistical significance.

## Results

### Patient and disease characteristics

A total of 90 neobladder cases were included in our study. They were stratified into 2 groups according to the surgical technique that was used for the orthotopic urinary diversion—the Studer and Y-pouch neobladder techniques as shown in Table 1. The median patient age was 62.6 (± 11) years. The Studer and Y-pouch neobladder techniques were performed in 54 (66.6%) and 36 (33.4%) patients, respectively. No severe intraoperative complications or deaths were reported. The mean operative time for the Studer technique was 290 min (IQR 242.5–350 min) and that for the Y-pouch technique was 300 min (IQR 271.2–335 min) (*p* = 0.826). The mean amount of blood loss (1000 mL, IQR 800–2000 mL for the Y-pouch technique vs. 1300 mL, IQR 1000–1650 mL for the Studer technique) was not statistically significant (*p* = 0.149).

**Table 1:** Tumor characteristics and perioperative outcomes of MIBC patients undergoing radical cystectomy with a neobladder via the Studer and Y-pouch techniques

Characteristics	Studer neobladder (*n* = 54)	Y-pouch neobladder (*n* = 36)	Total (*n* = 90)	*P* value
Age, mean (± SD)	62.5 (± 10.7)	62.7 (± 11.9)	62.6 (± 11)	0.957
Gender, *n* (%)				
Male	54 (100)	28 (77.8)	82 (91.1)	0.021
Female	0 (0)	8 (22.2)	8 (8.9)	
Presentation				
Gross hematuria	46 (85.2)	36 (100)	82 (91.1)	0.406
Microscopic hematuria	4 (7.4)	0 (0)	4 (4.4)	
No hematuria	4 (7.4)	0 (0)	4 (4.4)	
Time to radical cystectomy (days)				
Median (IQR)	53 (18.5, 78)	69 (63.2, 109.5)	65 (42, 92)	0.05
pT stage, *n* (%)				
2	40 (74)	28 (77.8)	68 (75.5)	0.199
3	12 (22.2)	2 (5.6)	14 (15.6)	
4	2 (3.7)	6 (16.7)	8 (8.9)	
pN stage, *n* (%)				
0	48 (88.9)	32 (88.9)	80 (88.9)	0.573
1	6 (11.1)	2 (5.6)	8 (8.9)	
2	0 (0)	2 (5.6)	2 (2.2)	
Grading				
Low grade	2 (3.7)	0 (0)	2 (2.2)	
High grade	52 (96.3)	36 (100)	88 (97.8)	
BMI				
Mean (± SD)	23.2 (± 2.8)	22.4 (± 2.7)	22.9 (± 2.8)	0.398
Baseline Cr (mg/dL)	1.02 (± 0.6)	1.00 (± 0.7)	1.01 (± 0.6)	0.23
Operative time				
Median (IQR)	290 (242.5, 350)	300 (271.2, 335)	290 (255, 340)	0.826
Cystectomy time				
Median (IQR)	140 (120, 150)	130 (120, 157.5)	140 (120, 150)	0.797
Neobladder time				
Median (IQR)	150 (132.5, 200)	155 (150, 185)	155 (145, 200)	0.78
Blood loss (mL)				
Median (IQR)	1300 (1000, 1650)	1000 (800, 2000)	1200(1000,1700)	0.149
Postoperative chemotherapy				0.166
No	8 (14.8)	12 (33.3)	20 (22.2)	
Yes	46 (85.2)	24 (66.7)	70 (77.8)	
Clean intermittent catheterization				0.064
Yes	16 (29.6)	2 (5.6)	18 (20)	
No	38 (70.4)	34 (94.4)	72 (80)	
Cystostomy				< 0.001
No	0 (0)	34 (94.4)	34 (37.8)	
Yes	54 (100)	2 (5.6)	56 (62.2)	
Catheterization days				< 0.001
Median (IQR)	21 (21, 21)	13.5 (10.2, 14)	21 (14, 21)	

*IQR* interquartile range, *BMI* body mass index

### Postoperative outcomes and complications

**Fig. 2:**
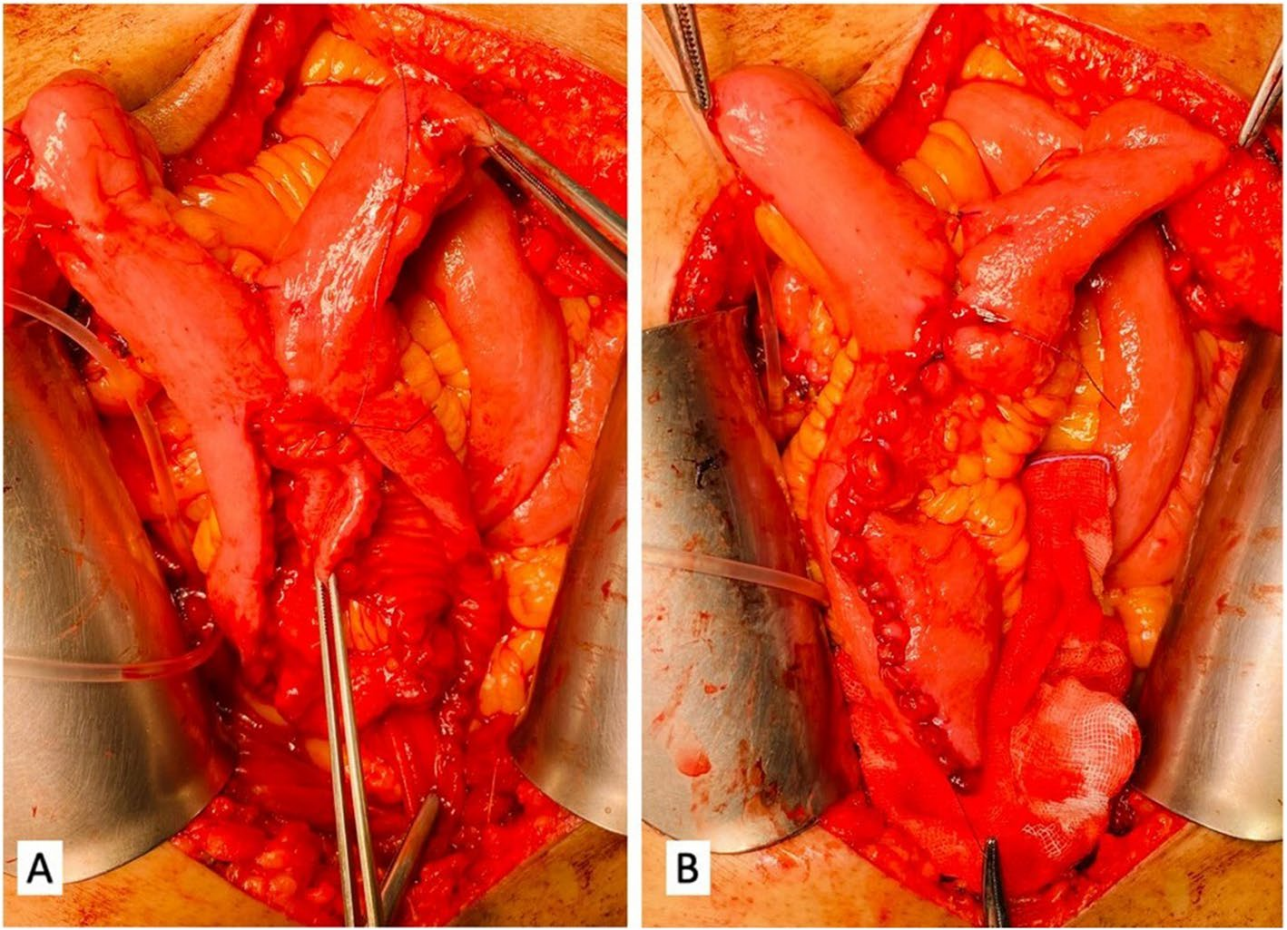
Small bowel suturing of the Y-pouch ileal neobladder. **A** Medial edges and **B** lateral edges of the ileal loop were sutured

The perioperative and histopathological data of the patients were stratified according to the neobladder surgical technique used and are summarized in Table 2. In all the cases, the operative technique was technically successful (Fig. 2). At 30 days postoperatively, the Clavien-Dindo classification of surgical complications revealed grade-2 urinary infections in two patients (5.6%) and six patients (11.1%) for the Y-pouch and Studer techniques, respectively. Late complications were reported in 4 (11.1%) and 18 patients (44.4%) in whom the Y-pouch and the Studer techniques were used, respectively (*p* = 0.062). The pouch calculi were detected in 2 cases (3.7), metabolic complication in 8 cases (14.8%), and uretero-intestinal anastomosis stricture in 6 cases (11.1%) in the Studer group. Y-pouch group exhibited uretero-intestinal anastosis stricture in 2 cases (5.6%) without pouch calculi and metabolic complications.

**Table 2:** Postoperative outcomes and complications

Characteristics	CDC	Studer neobladder technique (*n* = 54)	Y-pouch technique (*n* = 36)	*P* value
Short-term (≤ 30 days) complications				0.509
Complication category				
1. Gastrointestinal		18 (33.3)	11(30.1)	0.31
Ileus	II	16 (29.6)	10 (27.8)	0.28
Leakage from uretero-intestinal anastomosis	II	2 (3.7)	1 (2.8)	0.36
2. Genitourinary		14 (25.9)	10 (27.8)	0.34
UTI with fever	II	12 (22.2)	8 (22.2)	0.36
Urosepsis	II	2 (3.7)	2 (5.6)	0.41
3. Cardiovascular		3 (5.6)	1 (2.8)	0.22
Atrial fibrillation	II	3 (5.6)	1 (2.8)	0.22
4. Hematological and vascular		7 (13.0)	5 (13.9)	0.32
Postoperative ES transfusion	II	2 (3.7)	1 (2.8)	0.36
Fresh frozen plasma transfusion	II	3 (5.6)	2 (5.6)	0.23
Deep vein thrombosis	II	2 (3.7)	1 (2.8)	0.36
5. Pulmonary		4 (7.4)	3 (8.3)	0.35
Pulmonary infection		4 (7.4)	3 (8.3)	0.35
6. Wound		7 (13.9)	4 (11.1)	0.43
Infection	II	2 (3.7)	1 (2.8)	0.36
Superficial wound dehiscence	II	5 (9.3)	3 (8.3)	0.32
Intermediate-term (31–90 days) complications				0.192
1. Gastrointestinal		3 (5.6)	2 (5.6)	0.32
Constipation	II	3 (5.6)	2 (5.6)	0.32
2. Genitourinary		5 (9.3)	3 (8.3)	0.13
Renal function decline	I	2 (3.7)	1 (2.8)	0.36
UTI with fever	II	2 (3.7)	1 (2.8)	0.36
Pouch calculi	IIIb	1 (1.9)	1 (2.8)	0.26
UUT obstruction conservative (mild HUN)	I	2 (3.7)	1 (2.8)	0.36
3. Miscellaneous		2 (3.7)	1 (2.8)	0.23
Electrolyte imbalance	I	2 (3.7)	1 (2.8)	0.23
Urinary function				
Daytime continence		36 (75)	26 (72.5)	0.89
Nighttime continence		26 (54.1)	20 (55.5)	0.96
Daytime pads used at 12 months				0.083
Mean (± SD)		1.1 (± 0.8)	0.7 (± 0.7)	
Nighttime pads used at 12 months				0.099
Median (IQR)		1 (1.1)	1 (0.1)	
Capacity (mL)				
Mean (± SD)		428.9 (± 25)	417.2 (± 30.6)	0.169
Post-micturition residual urine at 2 months (mL)				
Mean (± SD)		50.7 (± 18.2)	26.7 (± 11.9)	< 0.001

*CDC* Clavien-Dindo classification, *UTI* urinary tract infection, *UUT* upper urinary tract, *HUN*, hydroureteronephrosis, *IQR* interquartile range, *CKD* chronic kidney disease

Continence usually improved over time, and continence recovery within a mean of 6 months postoperatively (range 1–12 months) was observed in most patients, irrespective of the surgical technique that was used. Moreover, according to Table 2, nighttime continence was achieved in 20 (55.5%) and 26 (54.1%) patients that underwent the Y-pouch and Studer techniques, respectively (*p* = 0.96). The achieved mean capacity of the Y-pouch neobladder at the 12-month follow-up was 417.2 (± 30.6) ml, which is not significantly different compared to that of the Studer neobladder 428.9 (± 25) ml (*p* = 0.169).

**Fig. 3:**
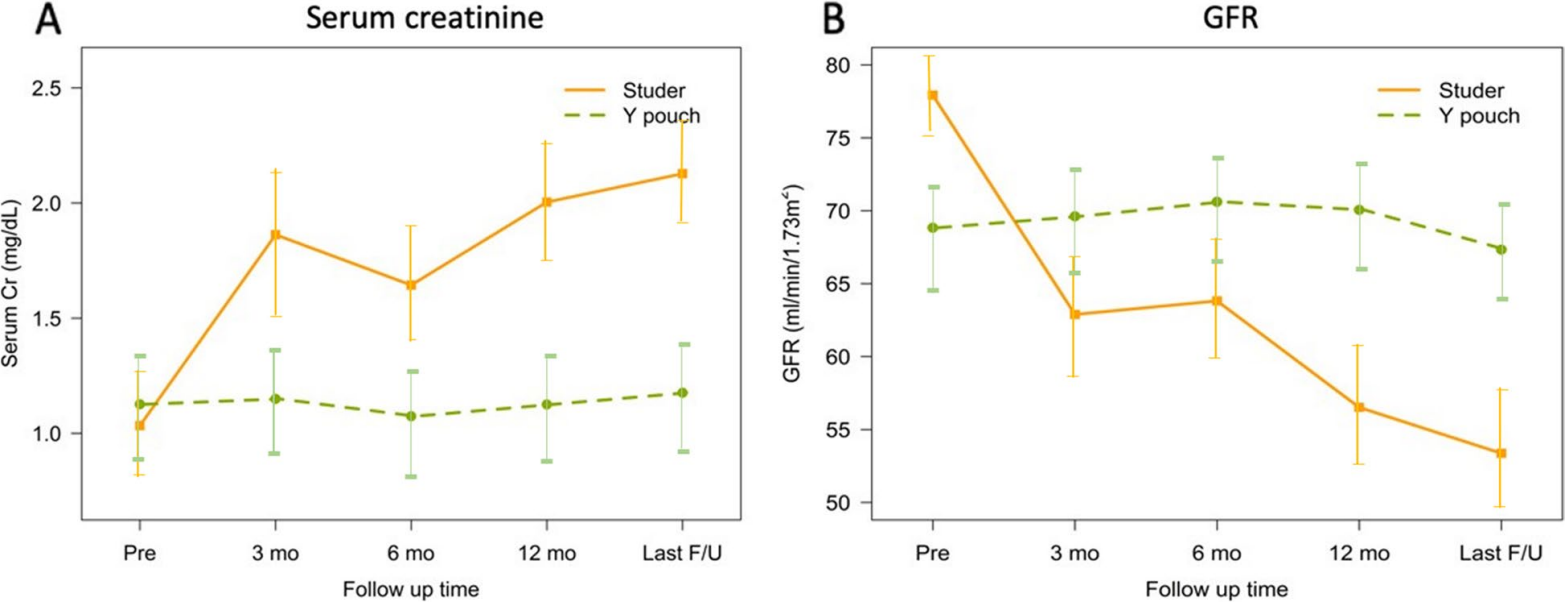
Comparison of changes in serum creatinine levels (**A**) and the estimated glomerular filtration rate (GFR) (**B**) with the standard deviation over time up to 12 months between the Y-pouch and Studer techniques

Finally, we evaluated the renal function by monitoring serum creatinine level and the estimated glomerular filtration rate (eGFR) preoperatively and at each follow-up visit until the completion of 12 months postoperatively. The serum creatinine value fluctuations at follow-up visits of all the patients according to the technique used are shown in Fig. 3A. The mean serum creatinine change in patients undergoing the Studer technique was + 0.23 mg/dL; a change of + 0.008 mg/dl was observed in patients receiving the Y-pouch technique. These indicated that there was a statistically significant difference in the mean serum change between the 2 groups (*P* = 0.0049) (Fig. 3A). Interestingly, the eGFR declined progressively after surgery in the Studer neobladder group (Fig. 3B). We also found that the patients who underwent the Studer technique exhibited a significant increase in serum creatinine levels and lower eGFRs at the 12-month follow-up. In our study, the mean eGFR change was − 5.5 mL/min/1.73 m^2^ in the patients who received a Studer neobladder and − 0.3 mL/min/1.73 m^2^ in those who received a Y-pouch neobladder. A statistically significant difference in the mean 12-month eGFR change was detected between the 2 groups (*p* = 0.007).

### Urodynamics outcome

Multi-channel UDS measurement is performed in our patients 12 months after radical cystectomy. In the study, urodynamic studies were conducted in 50% of patients in both the Studer neobladder group (27/54) and the Y-pouch neobladder group (18/36). The mean preurodynamic PVR was 46 mL (range 0–90 mL) in the Studer neobladder and 20 mL in the Y-pouch technique. None of these patients exhibited detrusor (neobladder) overactivity during the cystometry filling phase. The average compliance of both groups was normal (mean of 35.5 ml/cm H_2_O, range 28–52 for the Studer group and mean of 33 ml/cm H_2_O, range 30–43 for Y pouch), and most patients had > 30 ml/cm H_2_O compliance (80/90 patients). Urinary incontinence on provocative testing (Valsalva maneuver or coughing) was noted in each group with a mean ALPP of 52 cm H_2_O in the Studer group and 50 cm H_2_O in the Y-pouch group. Follow-up at 1 year had been completed by all patients (100%) in both groups (Table 3).

**Table 3:** Functional outcomes for patients undergoing open radical cystectomy and orthotopic neobladder for bladder cancer compared with 2 techniques

Characteristics	Studer neobladder technique (*n* = 27)	Y-pouch technique (*n* = 18)	*P* value
Neobladder maximal cystometric capacity (mL)	495 (400–628)	462 (380–600)	0.077
Neobladder compliance (mL/cmH_2_O)	35.5 (28–62)	33 (30–43)	0.069
*Q*_max_ (ml/s)	13.6 (12.8.–20.7)	14 (12.9–20.6)	0.32
PVR (mL)	46 (0–90)	20 (0–40)	0.06

## Discussion

ONB are used for urinary diversion after RC, and the use of different types has been reported. Although novel techniques have been described, controversies related to their efficacy and safety remain, and their use depends chiefly on surgeon preferences and experiences. Thus, the author has intended to perform the neobladder using a Y-pouch formation technique, which is safe and easy to construct and which also ensures a low pressure with an appropriate capacity. Furthermore, the continence function in both the day- and nighttime must be achieved [14]. The standard technique involves the creation of the Studer neobladder; however, other ileo-colonic neobladder techniques have also been used, such as the Karolinska-modified Studer neobladder, the University of Southern California-modified Studer neobladder, the N pouch, the W pouch, the modified Y-shaped, and other orthotopic neobladders for which the long-term outcomes are comparable in oncological and functional outcomes to standard Studer technique or ileal conduit [7, 15–18]. Although the Studer technique has significant advantages over the other alternatives. The Studer pouch has proved to be a reproducible technique, with good operative and functional outcomes obtained in multiple different centers; many of the largest series have reported daytime and nighttime continence rates reaching 85% and 70%, respectively [8]. However, no single neobladder technique has been shown to be clearly superior. Adherence to certain surgical guiding principles is paramount. The intracorporeal Studer pouch is a reproducible technique that provides favorable functional outcomes and is currently supported by the largest body of literature. Furthermore, the surgeons often select 1 or 2 additional techniques including a W pouch and Y pouch that are considered suitable and provide the best results and experienced that the Y-pouch technique has shown simple, fast, and less mucus production in the pilot cases. Specifically, in the Y-pouch group, the left side ureter is directly anastomosed to the left afferent limb of the Y-shaped neobladder, while in the Studer group, the left side ureter passes behind the mesentery. This observation emphasizes the significance of an in-place anastomosis between the left side ureter and the left afferent limb of the Y-pouch neobladder, which is considered one of the advantages of the Y-pouch technique over the Studer technique. Furthermore, this advantage makes the Y-pouch neobladder more suitable for laparoscopic or robotic surgery applications, providing increased comfort during the procedure.

The development of surgical techniques that are easy to perform and yield satisfactory long-term results is appreciated and necessary. Therefore, the authors performed a neobladder using a Y-pouch technique. A recent study also reported on this technique using a three-dimensional surgical postoperative pouch evaluation of the Y-pouch neobladder [18].

A simple shape, resembling that of the original bladder configuration, and ease of use are the main advantages of the Y-pouch. A 40-cm ileal segment is used, and a uretero-ileal anastomosis is performed between the spatulated ureters into the intestinal mucosa of both the Y limbs of the neobladder using the modified Lich-Gregoir technique, directly mucosa-to-mucosa without any anti-reflux method or by creating a chimney. The major concerns surrounding the anastomosis stricture explain why we do not follow the antireflux method; another reason is to ensure its ease of use with the intracorporeal neobladder. While a critical point to be aware of when performing an orthotopic neobladder reconstruction is the protection of the upper urinary tract, our technique did not account for this. Moreover, the meta-analysis showed that the overall incidence of vesicoureteral reflux was higher with direct anastomosis than anti-reflux anastomosis, and the rate of vesicoureteral reflux was not directly related to the impairment of RFn. The anti-reflux mechanism of ONB was positively associated with a higher incidence of significant ureteroenteric anastomotic stricture compared to the direct approach, which can lead to kidney damage and an increased risk of secondary surgical procedures [19].

However, compared to that in previous literature, the volume of blood loss in this study was higher, which was a point of concern. The issue of high blood loss in our study is indeed an important consideration. Radical cystectomy is a complex surgical procedure that involves meticulous dissection and removal of the bladder, along with lymph node dissection and urinary diversion. Despite our efforts to minimize blood loss during the procedure, certain factors may contribute to higher blood loss in our series. Such as the open approach and complexity of bladder cancer and the experience of surgeons.

The urethra strictures in 2 patients (11.1%) receiving the Y-pouch technique and 4 patients (14.8%) receiving the Studer technique required additional endoscopic treatment. A uretero-neobladder anastomosis was not found in any of the patients (0%) that underwent the Y-pouch technique; however, 1 patient that underwent the standard technique (3.7%) experienced this complication and was treated with additional open surgery. In our study, we observed a significant difference in the need for cystostomy between patients who underwent the Studer neobladder procedure and those who had the Y-pouch neobladder technique. The Studer neobladder, which involves constructing a reservoir using a longer segment of the ileum, often leads to increased mucus production, necessitating the use of cystostomy as an alternative method for urinary drainage. However, it is important to note that many centers do not utilize cystostomy after Studer pouch construction, which challenges the advantage previously suggested for the Y-pouch neobladder technique, and it is crucial to reevaluate the advantages of the Y-pouch neobladder. Although the absence of cystostomy in patients with the Y-pouch neobladder may not be a distinct advantage, it could still contribute to improved patient comfort and satisfaction. Furthermore, it is plausible that the lack of cystostomy in Y-pouch neobladder patients may have a favorable impact on postoperative urinary function and continence outcomes, without increasing the risk of inadequate drainage complications.

Metabolic acidosis occurring in the postoperative period and detected during the follow-up have been reported by several studies [7, 8]; for example, in 1 study, 48% of patients with an ileal neobladder required alkalizing treatment for an acidotic imbalance [7]. Moreover, the advantages of using a terminal ileal segment for an orthotopic urinary diversion to avoid metabolic complications have also been reported [20]. Ideally, the terminal ileal loop is the most suitable bowel segment for an ONB. No metabolic complications in our patients during either the early or the mid-term follow-up periods were observed. The creatinine and eGFR values between the 2 groups showed significant differences and better results for the Y-pouch neobladder.

Our hypothesis is explained by the lower urinary contact area of the small bowel. Theoretically, a smaller urinary contact area in the small bowel segment of the Y-pouch neobladder might result in a reduced urine absorption and also by less mucus production from this technique, and it has been suggested that the Y-pouch neobladder technique may lead to less mucus production compared to Studer approaches.

Moreover, patients with bladder substitution achieved daytime control more rapidly than those who underwent a radical prostatectomy, and stress urinary incontinence was reported rarely. Additionally, it mentions that stress urinary incontinence (involuntary leakage of urine during physical activities or exertion) was rarely reported in the context of bladder substitution [21]. Also, other results of continence in our cohort compared to other types of pouch such as N-shaped orthotopic ileal neobladder exhibited daytime continence rates were better than nighttime rates [22].

The rate of outlet obstruction by local recurrence was 2%, that of gross hematuria 1%, and that of entero-reservoir fistulas 1–2%. Daytime continence at 12 months was 92%, while nighttime continence was lower around 80%. Transient or permanent urinary retention was seen in 11–12% of male patients. In both series, long-term upper urinary tract safety was good. The risk of stenoses of the uretero-intestinal anastomosis with consecutive loss of renal function decreased with the introduction of non-refluxing implantation techniques. The rate of long-term metabolic complications remains low when adequate substitution with sodium bicarbonate is guaranteed in patients with impaired renal function. Patient selection and meticulous postoperative follow-up contributed to achieve good long-term results after cystectomy and orthotopic ileal neobladder substitution of the two large series of patients from the Universities of Ulm and Bern [23].

The current study analyzed the urodynamic findings of patients who underwent RC with ONB using a Y-shaped reconstruction compared to the Studer neobladder. The main findings were that Y-shaped neobladders met the requirement of a reservoir with high capacity, low pressure, appropriate voiding, and preservation of renal function compared to the Studer technique.

To date, there are no standard urodynamic values for the ileal neobladder. However, when compared to reference values of a native bladder in 50-year-old men, the urodynamic profile of our Y-shaped ICUD was consistent with a normo-capacity (300–600 ml) and normo-compliant bladder (low compliance less than 30 ml/cm H_2_O for non-neurogenic bladder) [24]. Although there is no widely accepted absolute normal compliance, it has been suggested that H_2_O values > 12.5–30 ml/cm represent the lower limit of normal. Our patients in this cohort study had UDS outcomes similar to those with a Studer neobladder in our study and other studies in terms of compliance and capacity [25–28], despite the absence of an afferent ileal segment an non-double-folding reconfiguration. Uroflowmetry data also showed mostly unobstructed maximum urinary flow (*Q*_max_ > 10 ml/s) with low PVR in most of the patients, suggesting satisfactory bladder emptying. There were some patients who need clean intermittent-self catheterization as compared to other reports with the rate ranging from 0 to 20% [27–32], but most of these patients had their urodynamic evaluation at 12 months, suggesting possible improvement of neobladder voiding over time. Interestingly, some patients in our series had neobladder wall over-activity during the cystometry filling phase, potentially causing transitory high internal pressure. This phenomenon, already reported in other series [20, 23], might be caused by the residual peristaltic activity of the ileum and requires further urodynamic assessment. Despite this, none of our patients had any deterioration in renal function emphasizing that the Y-shaped ONB appeared to be safe and satisfactory from the urodynamic point of view.

Pouch calculi occurred in patients with stapled neobladders but were absent in those with hand-sewn pouches. The common symptom was gross hematuria, and the calculi, typically less than 1 cm in diameter, were detected using KUB system CT scans during follow-up. Transurethral endoscopic lithotripsy successfully treated the calculi without functional complications. Calcium oxalate was the stone component in all cases. Although stapled neobladders may increase the risk of pouch calculi, a previous study reported comparable rates of stone formation to hand-sewn neobladders. Intermittent self-catheterization was the only variable predicting stone formation. We understand that the management of pouch calculi can vary depending on factors such as the size, composition, and location of the calculi. Surgical removal may be necessary in some cases, while preventive measures such as maintaining appropriate hydration, implementing dietary modifications, and regular surveillance may help minimize the risk of pouch calculi formation [33]. Furthermore, in addition to actions aimed to prevent infectious stones such as bladder and pouch irrigation, we recommended these patients undergo a full metabolic workup with targeted dietary changes and medical therapies.

However, there were some limitations of this study. It was a retrospective study that employed a small sample size of 36 patients who received the Y-pouch compared with the 54 patients who received the Studer neobladder. We also failed to present any long-term results (the follow-up period ended at 12 months postoperatively). However, the functional results and postoperative complication rate in the initial period associated with this technique were acceptable, and the trend of renal function parameters after an RC appeared better than that of the Studer technique. Future studies should include a larger number of patients and employ a prospective design to overcome the existing limitations. The Studer procedure and the Y-pouch technique presented in this study provide comparable perioperative outcomes in patients undergoing radical cystectomy with an ONB, at both the early and mid-term assessments. The Y-shaped neobladder meets the requirement of a reservoir with high capacity, low pressure, appropriate voiding, and preservation of renal function. Therefore, we propose the Y-pouch neobladder to be one of the alternative ileal neobladder techniques to the Studer neobladder. No questionnaires were used for sexual function or incontinence. Further prospective, randomized, controlled, comparative studies are needed to confirm its efficacy.

## Conclusion

Our study presented an efficacious Y-pouch technique for the surgical construction of an ONB with a complication rate as well as outcomes comparable to those of the standard Studer technique. In addition, the Y pouch is a technically easier procedure to perform than the Studer technique and is equally safe. The Y-pouch ileal neobladder is feasible and safe for use as the standard neobladder technique for urinary diversion in patients with bladder cancer undergoing radical cystectomy.

## Data Availability

The datasets generated during and/or analyzed during the current study are available from the corresponding author on reasonable request. Data is available on request from the corresponding author by email t13ers@hotmail.com and btanan@medicine.psu.ac.th.

## Abbreviations

RC: Radical cystectomy
ONB: Orthotopic neobladder
ERAS: Enhanced recovery after surgery
KUB: Kidney-urinary-bladder
UDS: Urodynamics study
PVR: Postvoid residual urine
ALPP: Abdominal leak-point pressure
eGFR: Estimated glomerular infiltration rate
